# Differential Effects of Snail-KO in Human Breast Epithelial Cells and Human Breast Epithelial × Human Breast Cancer Hybrids

**DOI:** 10.3390/ijms26157033

**Published:** 2025-07-22

**Authors:** Silvia Keil, Thomas Dittmar

**Affiliations:** Immunology and Tumor Biology, Center for Biomedical Education and Research (ZBAF), Witten/Herdecke University, 58448 Witten, Germany; silvia.keil@uni-wh.de

**Keywords:** cell–cell fusion, breast cancer, epithelial-to-mesenchymal transition, mixed/hybrid E/M phenotype

## Abstract

Snail and Zeb1 have been suggested as markers for the hybrid/mixed epithelial (E)/mesenchymal (M) state of cancer cells. Such cancer cells co-express E- and M-specific transcripts and possess cancer stem cell properties. M13HS-2/-8 tumor hybrid clones derived from human M13SV1-EGFP-Neo breast epithelial cells and human HS578T-Hyg breast cancer cells exhibited co-expression of Snail and Zeb1. To explore the impact of Snail on stemness/epithelial-to-mesenchymal transition (EMT)-related properties in M13HS-2/-8 tumor hybrid clones, Snail was knocked out (KO) using CRISPR/Cas9. Mammosphere formation, colony formation, Western blot analyses, cell migration, and invasion assays were conducted for the characterization of Snail knockout cells. Interestingly, Snail-KO in M13SV1-EGFP-Neo cells resulted in the up-regulation of vimentin and N-cadherin, suggesting EMT induction, which was associated with a significantly enhanced colony formation capacity. In contrast, EMT marker pattern and colony formation capacities of M13HS-2/-8 Snail-KO tumor hybrid clones remained unchanged. Notably, the mammosphere formation capacities of M13HS-2/-8 Snail-KO tumor hybrid clones were significantly reduced. The migratory behavior of all Snail-KO cells was not altered compared with their wild-type counterparts. In contrast, M13HS-2 hybrids and their M13HS-2 Snail-KO variant exhibited a markedly enhanced invasive capacity. Therefore, Snail plays a role as a mediator of stemness properties rather than mediating EMT.

## 1. Introduction

Epithelial-to-mesenchymal transition (EMT) is a cell-biological program in which sessile epithelial cells undergo changes to acquire a motile mesenchymal phenotype [1,2,3,4,5]. Apart from its crucial role in embryogenesis and wound healing, EMT is also recognized as an important mechanism in cancer progression [1,2,3,4]. In this context, EMT marks the initial step of the metastasis cascade when cancer cells or cancer cell clusters disseminate from the primary tumor [1,2,3,4].

EMT is characterized by the down-regulation of epithelial-specific genes, such as E-cadherin, claudins, and occludins, and the up-regulation of mesenchymal-specific transcripts, including N-cadherin, vimentin, and fibronectin [1,2,3,4]. The loss of cell adhesion molecules and cell–cell contacts, along with the gain of motile properties, allows cancer cells or cancer cell clusters to detach from the primary tumor and initiate metastatic spread [1,2,3,4]. EMT is a complex process induced by well-known triggers such as transforming growth factor-β (TGF-β), cytokines, and hypoxia, and regulated by a core EMT regulatory network, consisting of EMT transcription factors, such as Snail and Zeb1, miRNA networks (miR-34 and miR-200), post-translational modifications, and alternative splicing [1,3,4,6,7].

For a long time, it was believed that cancer cells existed in either an epithelial (E) or mesenchymal (M) state, making them either sessile or motile. However, recent data have shown that cancer cells can also acquire a stable intermediate state known as a hybrid/mixed epithelial–mesenchymal (E/M) state [7,8,9,10,11,12,13], which is associated with cancer stem cell (CSC) properties [11,14,15]. The mechanisms through which cancer (stem) cells enter and maintain this hybrid/mixed E/M state are not yet clear, but mathematical modeling suggests that this state is likely regulated by the “miR-34-5p-Snail” and “miR-200c-3p-Zeb1” core EMT networks [7,9,13].

Kröger and colleagues demonstrated that E, E/M, and M subtypes of human mammary epithelial HMLER cells exhibit distinct expression patterns of epithelial and mesenchymal-specific genes, including Snail and Zeb1 [11]. HMLER cells with an epithelial phenotype were negative for Snail and Zeb1, whereas the expression of these two EMT transcription factors was highly up-regulated in HMLER cells with a mixed/hybrid phenotype [11]. In contrast, mesenchymal-like HMLER cells exhibited similar Zeb1 levels, but markedly lower Snail levels than E/M HMLER cells [11]. Interestingly, CRISPR/Cas9-mediated KO of Zeb1 together with forced Snail expression in HMLER cells was accompanied by an arrest in the mixed/hybrid E/M state and an increased inherent tumor initiation capacity [11], which is a hallmark of CSCs [16,17]. Thus, the EMT transcription factor Snail may play a role in determining the mixed/hybrid E/M state in HMLER cells, while Zeb1 may promote full EMT. However, other studies have shown that Zeb1, rather than Snail, maintained CSC properties [15,18,19]. For instance, depletion of Zeb1 suppressed stemness and colonization of pancreatic cancer cells [19]. Additionally, Zeb1-induced CSC properties in breast cancer cells were reduced after the expression of neurogenin-3, which is a gene known to be repressed by Zeb1 [15]. Conversely, knockdown of Snail reversed stemness and inhibited tumor growth in ovarian cancer cells [20]. Similarly, the stem/progenitor state of skin epithelial cells and carcinomas was maintained by Snail [21]. These findings suggest that Snail also plays a role in promoting stemness properties in (cancer) cells.

In a previous study, we demonstrated that M13HS tumor hybrids, derived from human M13SV1-EGFP-Neo breast epithelial cells and human HS578T-Hyg breast cancer cells, exhibited co-expression of Snail and Zeb1 [22,23]. These hybrids showed enhanced colony formation and mammosphere formation capacity [22], aligning with the known role of these transcription factors in regulating stemness properties of cancer cells. However, when Zeb1 expression was stably knocked out using CRISPR/Cas9 in M13HS tumor hybrids and parental HS578T-Hyg breast cancer cells, only moderate phenotypic changes were observed [23]. For example, M13HS-2 Zeb1-KO tumor hybrids showed significantly higher expression levels of the malignant human mammary stem cell marker aldehyde dehydrogenase 1 (ALDH1) but exhibited decreased colony and mammosphere formation capacities compared with wild-type M13HS-2 tumor hybrids [23]. Similarly, Zeb1-KO did not result in altered expression of EMT-related markers, such as re-induction of E-Cadherin expression in all Zeb-KO cells [23].

However, the role of Snail in human M13V1-EGFP-Neo breast epithelial cells, human HS578T-Hyg breast cancer cells, and their M13HS tumor hybrids has not yet been investigated. Snail is a well-known EMT transcription factor regulated by a complex signaling network, involving integrin-linked kinase (ILK), phosphatidylinositol 3-kinase (PI3-K), mitogen-activated protein kinases (MAPKs), glycogen synthase kinase 3-β (GSK-3β), nuclear factor kappa-light-chain-enhancer of activated B cell (NF-κB) pathways, receptor tyrosine kinase signaling cascades, and TGF-β/Smad signaling [6,24]. Snail is the first discovered and most significant transcriptional repressor of E-cadherin [24], and high Snail expression levels are linked to cancer progression and increased risk for tumor recurrence [24,25,26,27]. Therefore, this study aims to explore the role of Snail in the E, E/M, and M states of human M13V1-EGFP-Neo breast epithelial cells, human HS578T-Hyg breast cancer cells, and their M13HS tumor hybrids.

## 2. Results

### 2.1. Snail-KO in Human M13SV1-EGFP-Neo Breast Epithelial Cells Is Associated with the Induction of EMT

**Figure 1:**
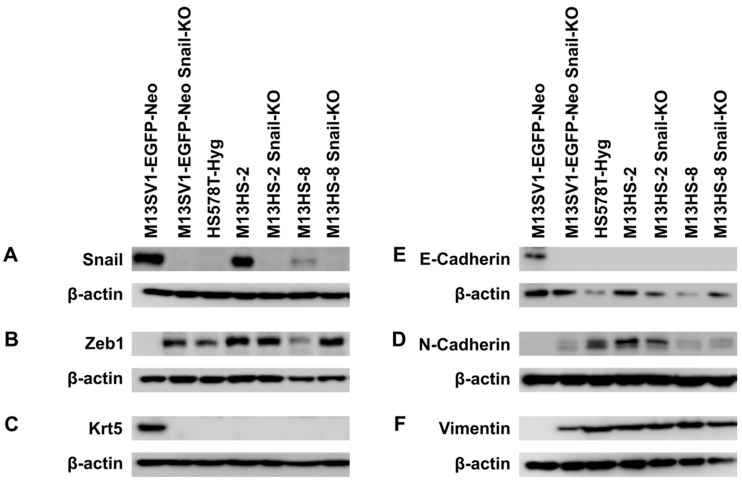
Stable CRISPR/Cas9-mediated Snail-KO in M13SV1-EGFP-Neo human breast epithelial cells results in EMT induction. (**A**) Snail. (**B**) Zeb1. (**C**) Cytokeratin 5. (**D**) E-cadherin. (**E**) N-cadherin. (**F**) Vimentin. Representative Western blot data of at least three independent experiments are shown.

The stable Snail-KO was generated using CRISPR/Cas9 in conjunction with puromycin-mediated selection of transfected cells. Growing clones were independently propagated, and the Snail-KO was validated through Western blot data (Figure 1) and Sanger sequencing (Figure S1).

A successful Snail-KO was observed in all analyzed M13SV1-EGFP-Neo and M13HS-2 and -8 clones. No Snail-KO was performed in HS578T-Hyg human breast cancer cells since they do not express this EMT transcription factor (Figure 1). Analysis of the EMT marker profile of M13HS-2/-8 tumor hybrids and their Snail-KO counterparts revealed no marked differences in the expression pattern of E-cadherin, N-cadherin, vimentin, and cytokeratin 5 (Figure 1A–F). However, M13HS-8 hybrids possessed lower Zeb1 expression levels compared with M13HS-2 hybrid cells (Figure 1B). Notably, Zeb1 expression levels were markedly higher in M13HS_8 Snail-KO cells than in M13HS-8 wild-type cells (Figure 1B). Interestingly, Snail-KO was associated with EMT induction in human M13SV1-EGFP-Neo Snail-KO breast epithelial cells. While parental M13SV1-EGFP-Neo breast epithelial cells exhibited a classical epithelial phenotype (E-cadherin and cytokeratin 5 positive, but Zeb1, N-cadherin, and vimentin negative), M13SV1-EGFP-Neo Snail-KO cells showed a completely opposite expression pattern (E-cadherin and cytokeratin 5 negative, but Zeb1, N-cadherin, and vimentin positive; Figure 1). This finding was rather unexpected since it indicates that the classical epithelial phenotype in M13SV1-EGFP-Neo cells is likely controlled by Snail.

### 2.2. M13SV1-EGFP-Neo Snail-KO Cells Demonstrate an Enhanced Capacity for Colony Formation

**Figure 2:**
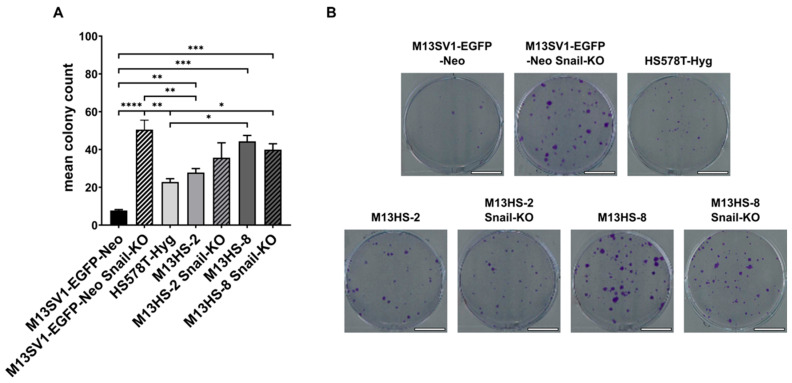
Stable CRISPR/Cas9-mediated Snail-KO in M13SV1-EGFP-Neo human breast epithelial cells resulted in a significantly increased colony formation capacity. (**A**) The mean ± S.E.M. values of at least three independent experiments are shown. (**B**) Representative images of colony formation assays. Bar = 10 mm. Statistical significance: one-way ANOVA and Tukey’s post hoc test. * = *p* < 0.0332, ** = *p* < 0.0021, *** = *p* < 0.0002, and **** = *p* < 0.0001.

Zeb1 has been proposed as a promoter of cancer cell stemness [9,11,28]. Therefore, we proceeded to assess the colony formation capability of both parental cells and Snail-KO cells (Figure 2).

The colony formation capacity of M13SV1-EGFP-Neo Snail-KO was significantly higher compared with wild-type M13SV1-EGFP-Neo cells (Figure 2A). Additionally, we observed a slight, though not significant, increase in colony formation capacity in M13HS-2 Snail-KO cells (Figure 2A). In contrast, M13HS-8 and M13HS-8 Snail-KO cells exhibited similar colony formation capacities (Figure 2A). Overall, these results support the hypothesis that Zeb1 may promote cell stemness.

### 2.3. The Mammosphere Formation Capacity of M13HS-2 and M13HS-8 Tumor Hybrids Is Markedly Reduced After Snail-KO

**Figure 3:**
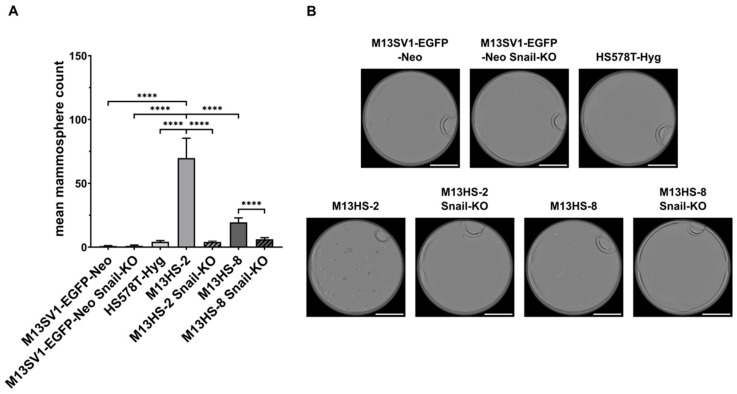
Stable CRISPR/Cas9-mediated Snail-KO resulted in a markedly diminished mammosphere formation capacity in M13HS-2-Snail-KO and M13HS-8-Snail-KO cells. (**A**) The mean ± S.E.M. values of at least three independent experiments are shown. (**B**) Representative images of mammosphere formation assays. Bar = 2 mm. Statistical significance: one-way ANOVA and Tukey’s post hoc test. **** = *p* < 0.0001.

The mammosphere formation assay is another in vitro method used to study potential stemness properties of cells. Consistent with previous findings [22,23], M13HS-2 and M13HS-8 tumor hybrids showed a markedly higher mammosphere formation capacity compared with M13SV1-EGFP-Neo and HS578T-Hyg parental cells (Figure 3A,B).

Interestingly, the high mammosphere formation capacity of M13HS-2 and M13HS-8 tumor hybrids was almost completely eliminated in their Snail-KO counterparts (M13HS-2: 70 ± 16 vs. M13HS-2 Snail-KO: 4 ± 1; M13HS-8: 19 ± 4 vs. M13HS-8 Snail-KO: 6 ± 1; Figure 3A). In contrast to M13HS-2 and M13HS-8 tumor hybrids, the mammosphere formation capacity of M13SV1-EGFP-Neo cells was unaffected after Snail-KO (Figure 3A). On average, only one mammosphere was found for each cell line in three independent experiments.

### 2.4. The Migratory Properties of Snail-KO Cells Are Only Slightly Altered Compared with Wild-Type Cells in a Scratch/Wound Healing Assay

**Figure 4:**
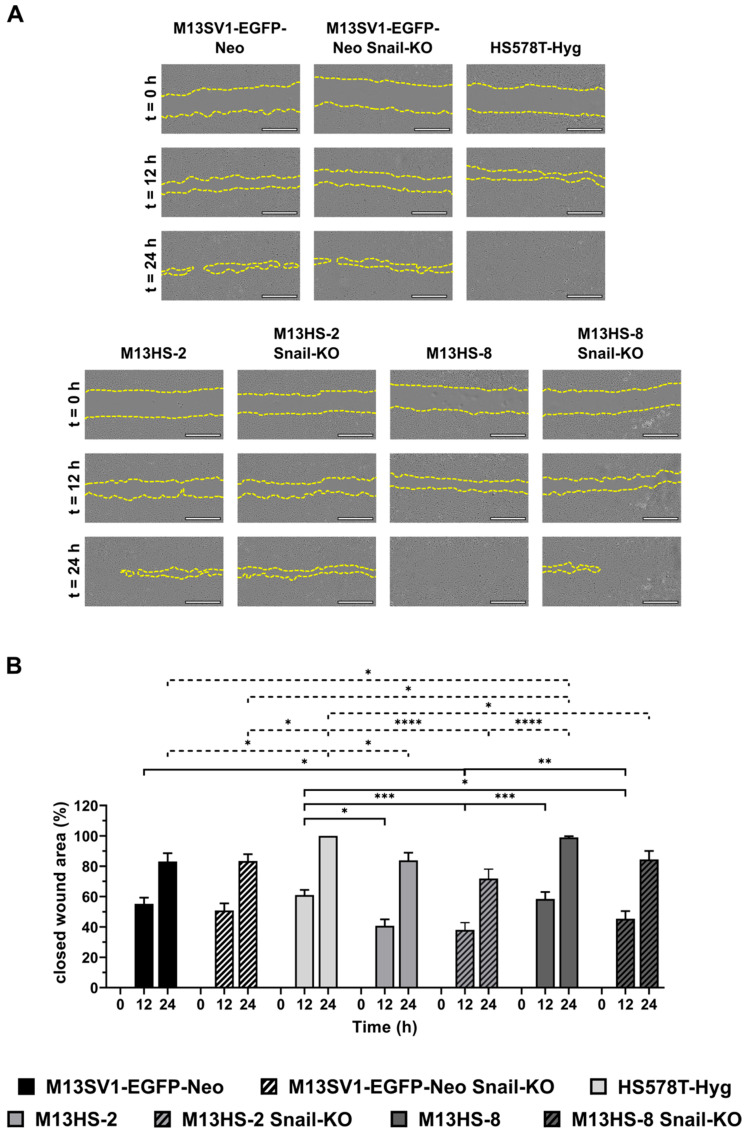
The migratory properties of Snail-KO M13SV1-EGFP-Neo breast epithelial cells and M13HS-2 and M13HS-8 hybrids were only slightly altered in a scratch-/wound-healing assay. (**A**) Representative images at t = 0 h, 12 h, and 24 h. The scratch/wound area is marked by a dashed yellow line. (**B**) Mean ± S.E.M. of the closed wound area of three independent experiments. Bar = 1 mm. Statistical significance was calculated using a two-way ANOVA and Tukey’s post hoc test. * = *p* < 0.0332, ** = *p* < 0.0021, *** = *p* < 0.0002, and **** = *p* < 0.0001.

To investigate the migratory behavior of Snail-KO cells compared with wild-type cells, we first performed a scratch-/wound-healing assay. The data are summarized in Figure 4, showing that M13SV1-EGFO-Neo Snail-KO cells and parental cells had similar migratory properties. In contrast, the migratory activities of M13HS-2 and M13HS-8 Snail-KO variants were lower, but not significantly lower, compared with M13HS-2 and M13HS-8 wild-type cells (Figure 4).

### 2.5. The Migratory Properties of Snail-KO Cells Are Only Slightly Altered Compared with Wild-Type Cells in a Transwell/Boyden Chamber Assay

**Figure 5:**
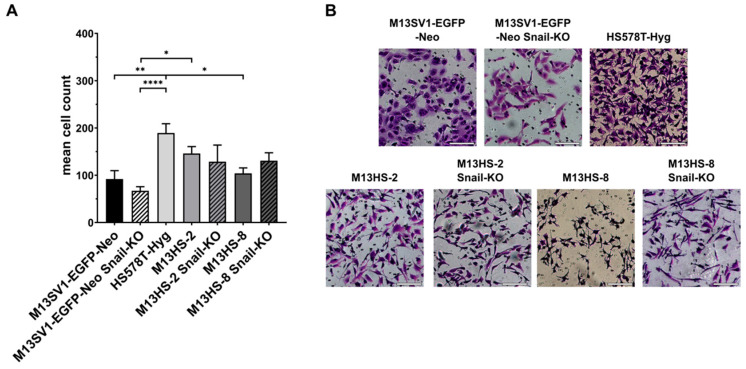
The migratory properties of Snail-KO M13SV1-EGFP-Neo breast epithelial cells and M13HS-2 and M13HS-8 hybrids were only slightly altered in the Transwell/Boyden chamber assay. (**A**) The mean ± S.E.M. values of at least three independent experiments are shown. (**B**) Representative images. Bar = 100 µm. Statistical significance: one-way ANOVA and Tukey’s post hoc test. * = *p* < 0.0332, ** = *p* < 0.0021, and **** = *p* < 0.0001.

In addition to the scratch-/wound-healing assay, we also conducted a Transwell/Boyden chamber assay to assess the migratory properties of wild-type cells and Snail-KO cells. The data obtained were relatively similar to the scratch-/wound-healing assay data. The highest locomotory activity was observed in HS578T-Hyg breast cancer cells (189 ± 20 cells), while M13SV1-EGFP-Neo breast epithelial cells had the lowest migratory activity among all wild-type cells (92 ± 18 cells; Figure 5A).

Consistent with the scratch-/wound-healing assay data, the migratory behavior of Snail-KO cells was only slightly different from wild-type cells. For example, M13SV1-EGFP-Neo breast epithelial cells and M13HS-2 hybrids showed a decrease in migratory activity in their Snail-KO variants (Figure 5A). Conversely, the migratory activity of M13HS-8 Snail-KO cells was slightly higher (131 ± 17 cells) compared with wild-type M13HS-8 hybrids (104 ± 12 cells; Figure 5A).

### 2.6. The Snail-KO in M13HS-2 Tumor Hybrids Is Associated with a Significantly Increased Invasion Capacity

**Figure 6:**
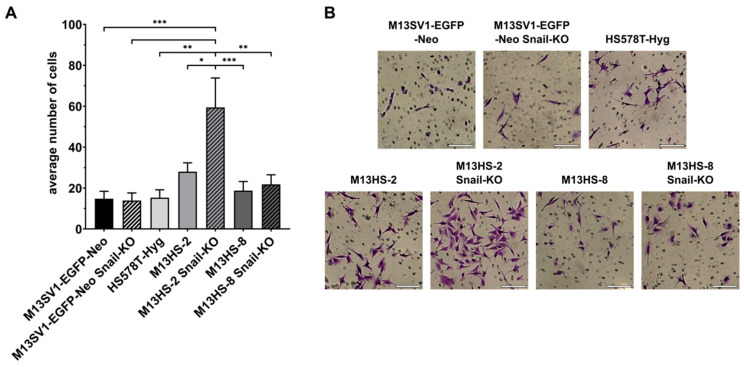
The invasive capacity of M13HS-2 cells was markedly enhanced after Snail-KO. (**A**) The mean ± S.E.M. values of at least three independent experiments are shown. (**B**) Representative images. Bar = 100 µm. Statistical significance: one-way ANOVA and Tukey’s post hoc test. * = *p* < 0.0332, ** = *p* < 0.0021, and *** = *p* < 0.0002.

Next, an invasion assay was performed to study the invasive capacity of wild-type and Snail-KO cells. Interestingly, the invasion capacities of M13SV1-EGFP-Neo, M13SV1-EGFP-Neo Snail-KO, HS578T-Hyg, M13HS-8, and M13HS-8 Snail-KO cells were rather comparable (Figure 6A), with the average number of invaded cells ranging between 14 ± 4 cells (M13SV1-EGFP-Neo Snail-KO) and 22 ± 5 cells (M13HS-8 Snail-KO) (Figure 6A).

In contrast, the invasion capacity of M13HS-2 hybrids (28 ± 4 cells) was markedly higher compared with the other parental cells (Figure 6A). Notably, M13HS-2 Snail-KO cells (59 ± 14 cells) possessed the highest invasion capacity of all cells (Figure 6A).

## 3. Discussion

In the present study, we investigated the role of the EMT transcription factor Snail and whether its knockout was associated with alterations in the E, E/M, and M states of the investigated cell lines.

The expression of the EMT transcription factor Snail in human M13SV1-EGFP-Neo breast epithelial cells remains ambiguous. As mentioned in the introduction, Snail was the first discovered and most important transcriptional repressor of E-cadherin [24]. However, human M13SV1-EGFP-Neo breast epithelial cells exhibit a classical epithelial phenotype (E-cadherin positive; negative for N-cadherin and vimentin) despite the expression of Snail as well as Twist and Slug [22], which are two other well-known EMT transcription factors and E-cadherin repressors [24]. Therefore, the observation that M13SV1-EGFP-Neo cells underwent a potential full EMT after Snail knockout was unexpected.

It is well known that Snail not only suppresses E-cadherin expression but also promotes transcription of Zeb1 mRNA, which is then translated to Zeb1 [29]. However, human M13SV1-EGFP-Neo breast epithelial cells do not express Zeb1. This may be related to the high miR-200c-3p expression levels in this cell line [23]. MiR-200c-3p is a known repressor of Zeb1 [7,9,29,30,31]. Therefore, it is possible that Snail induces the transcription of Zeb1 mRNA in M13SV1-EGFP-Neo cells and that Zeb1 translation is prevented by miR-200c-3p. However, Snail is also a well-known repressor of miR-200c-3p transcription [7,9,32,33]. Therefore, M13SV1-EGFP-Neo cells should not exhibit increased miR-200c-3p levels due to Snail expression. Moreover, the finding that the Snail knockout was associated with Zeb1 expression in M13SV1-EGFP-Neo cells may indicate that Snail acts as a Zeb1 repressor in this cell line. However, this is in contrast to the knowledge that Snail is a well-known promoter of Zeb1 expression [29]. Therefore, the finding that human M13SV1-EGFP-Neo breast epithelial cells exhibit a classical epithelial phenotype despite the expression of well-known EMT transcription factors such as Snail is most likely a cell line-specific phenomenon.

The data from the colony formation assay and mammosphere formation assay are challenging to interpret. M13SV1-EGFP-Neo Snail-KO cells showed a significantly enhanced colony formation capacity, but their mammosphere formation capacity remained unchanged compared with M13SV1-EGFP-Neo wild-type cells. On the other hand, the colony formation capacity of M13HS-2 Snail-KO and M13HS-8 Snail-KO cells was similar to that of their wild-type counterparts, but the mammosphere formation capacity of the Snail-KO cells was significantly reduced.

The finding that M13SV1-EGFP-Neo Snail-KO cells had a significantly enhanced colony formation capacity may be related to Zeb1 up-regulation, which has been suggested as a promoter of cancer cell stemness [9,11,28]. However, it remains unclear why the mammosphere formation capacity of M13SV1-EGFP-Neo Snail-KO cells remained unchanged despite Zeb1 expression. The finding that M13HS parental cells and M13HS Snail-KO cells had similar colony formation capacities might also be in agreement with the Zeb1 expression levels of the cells, which were not affected by Snail-KO. Indeed, we have previously shown that the colony formation capacity of M13HS Zeb1-KO variants was significantly decreased compared with their M13HS counterparts [23]. These findings may suggest that the colony formation capacity of (cancer) cells may be promoted by Zeb1. In contrast, the mammosphere formation capacity of M13HS-2 Snail-KO and M13HS-8 Snail-KO cells was significantly reduced. In this context, we have observed that the mammosphere formation capacity of M13HS-2 Zeb1-KO and M13HS-8 Zeb1-KO cells was also significantly reduced [23]. These findings may support the assumption that the capacity of (cancer) cells to form mammospheres is promoted by both Snail and Zeb1. Indeed, Snail and Zeb1 induced cell malignancy and a cancer stem cell phenotype in prostate cells [34]. However, it remains unclear whether M13HS-2 and M13HS-8 tumor hybrids exhibit a potential hybrid/mixed E/M state. Kröger and colleagues demonstrated that HMLER mammary cells with a hybrid/mixed E/M state co-expressed Snail and Zeb1 [11], supporting the hypothesis that M13HS-2 and M13HS-8 tumor hybrids may also show a hybrid/mixed E/M phenotype. Animal studies showed that HMLER mammary cells with a hybrid/mixed E/M state were more tumorigenic and formed about 10-fold larger tumors than implanted HMLER cells with either an E or M state [11]. Additionally, limited dilution assays revealed that the frequency of tumor-initiating CSCs in the E/M population was 122- and 3.7-fold higher than the corresponding CSC frequencies in E and M populations, respectively [11]. However, the potential tumorigenic capacity of M13HS-2 and M13HS-8 tumor hybrids has not yet been investigated. Nonetheless, the hypothesis that both Snail and Zeb1 may promote mammosphere formation could explain why M13SV1-EGFP-Neo parental cells and M13SV1-EGFP-Neo Snail-KO showed no mammosphere formation capacity. The parental cells express Snail but not Zeb1, while the M13SV1-EGFP-Neo Snail-KO expresses Zeb1 but not Snail. Thus, one of these two EMT transcription factors is still missing. In this context, it would be interesting to investigate whether M13SV1-EGFP-Neo parental cells overexpressing Zeb1 would have an enhanced capacity for mammosphere formation.

The migratory behavior of Snail-KO cells was not markedly altered compared with their wild-type counterparts, which was unexpected for M13SV1-EGFP-Neo Snail-KO cells. This was surprising due to the expression of Zeb1, N-cadherin, and vimentin, which likely indicate a potential EMT induction. EMT is the initial stage in cancer metastasis [1,35], and the EMT activator Zeb1 has been identified as a key factor in promoting metastasis formation in various types of cancer, including hepatocellular carcinoma and pancreatic, prostate, colorectal, and breast cancers [18,19,31,36,37,38]. For instance, Zeb1 promoted the migration of hepatocellular carcinoma cells [37] and enhanced the transendothelial migration of prostate cancer cells [38]. In breast cancer cells, the interaction of Zeb1 and CD44s increased tumor-sphere initiation capacity, drug resistance, and tumor recurrence [18]. CD44 is a multifunctional cell surface adhesion receptor highly expressed in many cancers, promoting migration and invasion processes involved in metastasis [39]. Therefore, Zeb1, along with CD44, promotes cancer cell migration. M13SV1-EGPF-Neo Snail-KO cells are CD44 positive and, therefore, an enhanced migratory activity was expected. However, the reason for this unexpected finding is currently unknown. It is possible that the EMT process was incomplete. In this context, it must be considered that the EMT status of cells cannot be determined based on a few molecular markers [5]. Instead, cellular properties, along with molecular markers, should be used to define the EMT status [5].

The invasion assay results showed that M13HS-2 tumor hybrids exhibited the highest invasion capacity, and M13HS-8 tumor hybrids showed the second highest invasion capacity among all parental cells, which is consistent with previous findings [23]. Interestingly, the invasion capacity of M13HS-2 Snail-KO cells was the highest among all cells, while the invasion capacity of M13HS-8 Snail-KO cells was similar to that of M13HS-8 tumor hybrids. We hypothesize that the increased invasive capacities of M13HS-2 and M13HS-8 tumor hybrids support the idea that tumor hybrids often have altered properties, such as an enhanced metastatic capacity or drug resistance [40,41,42,43]. However, the reason why M13HS-2 Snail-KO tumor hybrids, but not M13HS-8 Snail-KO tumor hybrids, exhibited significantly enhanced invasion capacity compared with their wild-type counterparts remains unclear. Therefore, it is not possible to provide a suitable explanation for these results without more in-depth analysis methods, such as characterizing the transcriptome of M13HS Snail-KO cells and their parental counterparts.

## 4. Materials and Methods

### 4.1. Cell Culture

M13SV1-EGFP-Neo, HS578T-Hyg, and M13HS-2 and M13HS-8 cells were generated and cultivated as previously described [23,44,45]. In short, M13SV1-EGFP-Neo human breast epithelial cells were derived from M13SV1 cells (kindly provided by James Trosko, Michigan State University, East Lansing, MI, USA [46]) through stable transfection with the pEGFP-Neo vector [45]. HS578T-Hyg human breast cancer cells were created from HS578T cells (HTB 126; LGC Standards GmbH, Wesel, Germany) via stable transfection with the pKS-Hyg plasmid. M13HS-2 and M13HS-8 hybrid cells were the result of spontaneous fusion events between M13SV1-EGFP-Neo cells and HS578T-Hyg cells [45]. All cells were grown in RPMI 1640 media (PAN Biotech GmbH, Aidenbach, Germany) supplemented with 10% fetal bovine serum (PAN Biotech GmbH, Aidenbach, Germany) and 100 U/mL penicillin/0.1 mg/mL streptomycin (PAN Biotech GmbH, Aidenbach, Germany). Additional supplements were added to the medium based on the specific cell line. For M13SV1-EGFP-Neo cells, supplements included 0.5 ng/mL recombinant human epidermal growth factor, 5 µg/mL human recombinant insulin, 0.5 µg/mL hydrocortisone, 4 µg/mL human transferrin, 10 nM β-estrogen, and 400 µg/mL G418 (all supplements were purchased from Merck KGaA, Darmstadt, Germany). HS578T-Hyg was supplemented with 200 µg/mL hygromycin B (Pan Biotech, Aidenbach, Germany). M13HS-2 and M13HS-8 hybrid cells were supplemented with 400 µg/mL G418 (Merck KGaA, Darmstadt, Germany) and 200 µg/mL hygromycin B (Pan Biotech, Aidenbach, Germany). All cells were cultured in a humidified atmosphere at 37 °C and 5% CO_2_.

### 4.2. Generation of Snail-Knockout (KO) Cells

Snail-KO variants of M13SV1-EGFP-Neo, M13HS-2, and M13HS-8 cells were generated through CRISPR/Cas9-mediated gene editing as described by Merckens and colleagues [23]. The Snail spacer guide RNA sequence (5′-GGG ACT CTC CTG GAG CCG AA-3′) was taken from the study by the authors of [11]. Sense and antisense oligonucleotides (sgRNA_Snail_fwd: 5′-AAA CTT CGG CTC CAG GAG AGT CCC-3′, sgRNA_Snail_rev: 5′-CAC CGG GAC TCT CCT GGA GCC GAA-3′; Thermo Fisher Scientific, Wesel, Germany) were annealed and ligated into the BbSI-digested pX330-U6-Chimeric_BB-CBh-hSpCas9-P2A-PuroR (CRISPR/Cas9-sgRNA) vector [23]. This vector was created by inserting a FseI-p2A-PuroR-bGH Poly(A)-NotI fragment from pcDNA3.1_iCre-T2A-mCherry-p2A-PuroR into Fse1/NotI restricted pX330-U6-Chimeric_BB-CBh-hSpCas9 plasmid (pX330-U6-Chimeric_BB-CBh-hSpCas9 was a gift from Feng Zhang; Addgene plasmid #42230; http://n2t.net/addgene:42230, accessed on 1 February 2020; RRID: Addgene_42230). The correct cloning of CRISPR/Cas9-sgRNA was confirmed through Sanger sequencing (Eurofins Genomics, Ebersbach, Germany) and using SnapGene 5.3.1 software (Dotmatics, Boston, MA, USA). The CRISPR2/Cas9-sgSnail vector was amplified in NEB 10β bacteria (New England Biolabs GmbH, Frankfurt am Main, Germany) and purified using the Nucleospin^®^ Plasmid Transfection-grade kit following the manufacturer’s instructions (Macherey-Nagel GmbH, Düren, Germany). The jetOPTIMUS^®^ DNA transfection reagent (Polyplus, Illkirch, France) was used to transfect M13SV1-EGFP-Neo, M13HS-2, and M13HS-8 cells with the CRISPR/Cas9-sgRNA vector as per the manufacturer’s protocol. Twenty-four hours post-transfection, 2 µg/µL of puromycin (Thermo Fisher Scientific, Wesel, Germany) was added to the culture medium for up to 72 h to eliminate non-transfected cells. The surviving cells were cultured until they formed colonies with an average diameter of 2 to 3 mm. Single cell-derived colonies were picked by soaking 3 mm Whatman paper discs (Merck KGaA, Darmstadt, Germany) with trypsin 0.25%/1 mM EDTA (PAN Biotech GmbH, Aidenbach, Germany) and then transferring them to 96-well plates (Sarstedt AG & Co. KG, Nümbrecht, Germany) for further propagation. 

Genomic DNA was extracted from cells (both parental cells and Snail-KO clones) following the NucleoSpin^®^ Tissue DNA kit manual (Macherey & Nagel, Düren, Germany). Initially, the CRISPR/Cas9 edited gene sequence was amplified using PCR (T7E1_Snail_fwd: 5′-AGT TTA CCT TCC AGC AGC CC-3′; T7E1_Snail_rev: 5′-AGA TCC TTG GCC TVA GAG AG-3′; Thermo Fisher Scientific, Wesel, Germany). Subsequently, the PCR product was subjected to Sanger sequencing (Eurofins Genomics, Ebersbach, Germany) using the following sequencing primers (Seq_Snail_fwd: 5′-TCG CTG CCA ATG CTC ATC TG-3′; Seq_Snail_rev: 5′-GCC TCC AAG GAA GAG ACT GAA G-3′; Thermo Fisher Scientific, Wesel, Germany). Sequences were analyzed with SnapGene 5.3.1 software (Dotmatics, Bishops Stortford, UK). Successfully edited CRISPR/Cas9 Snail-KO clones of M13SV1-EGFP-Neo, M13HS-2, and M13HS-8 cells were combined and expanded for further studies.

### 4.3. Western Blot Analysis

All cells were harvested using the standard protocol with trypsin 0.25%/1mM EDTA (PAN Biotech GmbH, Aidenbach, Germany) and washed once with PBS. Total cell lysates were prepared for Western blot analysis by adding 10 µL of 3× Laemmli sample buffer to 20 µL of cell suspension (2 × 10^5^ cells/20 µL PBS) and then incubating for 10 min at 95 °C. Depending on the molecular weights of the target proteins (Table 1), the samples were separated by either 10% or 12% sodium dodecyl sulfate–polyacrylamide gel electrophoresis (SDS-PAGE) and transferred to an Amersham^™^ Protran^™^ 0.45 µm nitrocellulose blotting membrane (Merck KGaA, Darmstadt, Germany). The membranes were blocked either with 5% bovine serum albumin (BSA) or 5% (*w*/*v*) non-fat milk powder in Tris-buffered saline with 0.1% (*v*/*v*) Tween 20 (TBS-T), depending on the used antibodies (Table 1) and the manufacturer’s recommendations. Finally, bands were visualized using the Pierce ECL Western blot substrate (Thermo Fisher Scientific, Wesel, Germany), according to the manufacturer’s protocol and the Aequoria Macroscopic Imaging System (Hamamatsu Photonics Germany, Herrsching am Ammersee, Germany).

### 4.4. Colony Formation Assay

The colony formation assay was conducted as described by the authors of [23,47,48]. In short, 500 cells were seeded per well in 6-well plates and cultured for 10 days in a humidified atmosphere at 37 °C and 5% CO_2_. Following incubation, the media was aspirated, and then the cells were rinsed twice with PBS and fixed with 4% paraformaldehyde solution (Agilent Technologies Deutschland GmbH, Waldbronn, Germany). The fixed colonies were then stained with a 0.5% crystal violet solution (Merck KGaA, Darmstadt, Germany) for 30 min at room temperature. Finally, the 6-well plates were thoroughly washed with water and air-dried.

### 4.5. Mammosphere Formation Assay

The mammosphere formation assay was conducted as described by the authors of [23,47,48]. In summary, the cells were gently resuspended in mammosphere formation medium (500 cells/200 µL medium) and then seeded into ultra-low-attachment 96-well plates (Sarstedt AG & Co KG, Nümbrecht, Germany). The mammosphere formation medium is a combination of two different media (I and II) in a 1:4 ratio. Medium I consists of DMEM/F12 medium (Pan Biotech GmbH, Aidenbach, Germany), 6.6% B27 supplement (Thermo Fisher Scientific, Wesel, Germany), 20 ng/mL human recombinant FGF, 20 ng/mL human recombinant EGF, and 0.39 µg/mL hydrocortisone. FGF, EGF, and hydrocortisone were obtained from Merck KgaA, Darmstadt, Germany. Medium II is a mixture of Methocult H4100 (Stem Cells Technologies, Cologne, Germany) and DMEM (Pan Biotech GmbH, Aidenbach, Germany) in a 2:3 ratio. The mammospheres were then incubated for 10 days in a humidified atmosphere at 37 °C and 5% CO_2_. The Incucyte^®^ SX5 Live-Cell Analysis System (Sartorius, Göttingen, Germany) was used to determine the mammosphere formation capacity, with mammospheres smaller than 60 µm in diameter being excluded from the analysis.

### 4.6. Scratch-/Wound-Healing Assay

The scratch-/wound-healing assay was conducted as described by the authors of [23]. In brief, the cells were seeded in triplicates (2 × 10^5^ cells/well) in a 24-well plate (Sarstedt AG & Co KG, Nümbrecht, Germany) and cultivated in a humidified atmosphere at 37 °C and 5% CO_2_ until they reached 100% confluency, which typically took 24 to 48 h depending on the cell line used. A 10 µL pipette tip was used to create the scratch/wound. The cells were then washed once with PBS to remove dead cells and cell debris. Subsequently, 1.5 mL of fresh media was added, and the 24-well plate was placed in the Incucyte^®^ SX5 Live-Cell Imaging system (Sartorius Lab Instruments GmbH, Göttingen, Germany). Transmission light images were captured every four hours for a total of 24 h at 37 °C and 5% CO_2_. The closure of the scratch/wound was determined using the Fiji software (Image J; Version 2.14.0/1.54f; https://Fiji.sc). The scratch/wound size at t = 12 and 24 h was calculated relative to the scratch/wound size at t = 0 h, which was set to 100%.

### 4.7. Transwell/Boyden Chamber Assay and Invasion Assay

The Transwell/Boyden chamber migration and invasion assays were conducted as described by the authors of [23]. For the Transwell/Boyden chamber migration assay, 6.5 × 10^4^ cells were seeded in the upper compartment of the Transwell insert (diameter 6.5 mm, 8 µm pore size; Becton Dickenson, Heidelberg, Germany) and placed in a 24-well plate (Sarstedt AG & Co KG, Nümbrecht, Germany) in 100 µL of serum free media. The lower compartment was filled with 250 µL of complete media.

For invasion studies, 100 µL of a 1:4 dilution of 4 °C cold Geltrex (Thermo Fisher Scientific, Wesel, Germany) with 4 °C cold PBS was used to coat the Transwell inserts. The inserts were then incubated for 60 min at 37 °C. Next, 6.5 × 10^4^ cells in 100 µL of serum-free media were carefully seeded on top of the polymerized Geltrex matrix. The lower compartment was filled with 250 µL of complete media.

Both assays were conducted for 24 h at 37 °C and 5% CO_2_. After incubation, the Transwell insert was removed, and any cells remaining in the upper compartment were gently removed with a cotton swab. The transmigrated cells were fixed with 4% paraformaldehyde (Agilent Technologies Deutschland GmbH, Waldbronn, Germany) for 15 min at room temperature. The fixed cells were then washed twice with PBS and stained with 0.5% crystal violet staining solution (Merck KGaA, Darmstadt, Germany) for 1 h at room temperature. Finally, the fixed and stained cells were thoroughly washed with water and air-dried. For each experiment, six randomly chosen fields were captured using an inverted microscope (Leica DM IRB; Leica, Wetzlar, Germany) and the Zeiss Labscope software (Carl Zeiss Microscopy GmbH, Jena, Germany). The Cell Counter plugin of the Fiji software (Image J; Version 2.14.0/1.54f; https://Fiji.sc) was utilized for quantifying the transmigrated cells.

### 4.8. Statistical Analysis

The GraphPad PRISM software 8.4.3 (graphpad.com) was utilized for statistical analysis (https://www.graphpad.com, accessed on 1 February 2020). Detailed descriptions of the statistical tests used and corresponding *p*-values can be found in the appropriate figure legends.

## 5. Conclusions

Based on the data obtained in this study, we conclude that the EMT transcription factor Snail is involved in mediating certain stemness capabilities of (cancer) cells. However, our results, both from this study and those recently published [23], further suggest that Zeb1 is also involved in mediating the stemness capabilities of cancer cells. For example, the mammosphere formation ability of M13HS tumor hybrids was significantly decreased after both Snail-KO and Zeb1-KO [23], highlighting the requirement of both EMT transcription factors for mammosphere formation. However, it is unclear why the ability of the cells to form colonies was not reduced after Snail-KO if Snail is involved in mediating stemness properties. Our data suggest that the ability of cells to form colonies is more dependent on Zeb1. Therefore, the extent to which Snail and Zeb1 are involved in the expression of the hybrid/mixed E/M phenotype cannot be conclusively determined. This should be investigated in further studies.

## Figures and Tables

**Table 1:** Used antibodies.

Antibody	Clone; Catalog Number	Manufacturer
Anti-β-Actin (mouse monoclonal)	Clone AC-15; #A5441	Merck KGaA, Darmstadt, Germany
E-cadherin (rabbit monoclonal)	Clone 24E10; #3195S	Cell Signaling Technology Europe B.V., Frankfurt am Main, Germany
Cytokeratin-5 (rabbit monoclonal)	Clone D4U8Q; #25807S	Cell Signaling Technology Europe B.V., Frankfurt am Main, Germany
N-cadherin (mouse monoclonal)	Clone 13A9; #14215S	Cell Signaling Technology Europe B.V., Frankfurt am Main, Germany
Snail (rabbit monoclonal)	Clone C15D3; #3879S	Cell Signaling Technology Europe B.V., Frankfurt am Main, Germany
Vimentin (rabbit polyclonal)	#3932S	Cell Signaling Technology Europe B.V., Frankfurt am Main, Germany
Zeb1 (rabbit monoclonal)	Clone D808D3; #3396S	Cell Signaling Technology Europe B.V., Frankfurt am Main, Germany
Anti-mouse IgG, HRP-linked (horse polyclonal)	#7076S	Cell Signaling Technology Europe B.V., Frankfurt am Main, Germany
Anti-rabbit IgG, HRP-linked (horse polyclonal)	7074S	Cell Signaling Technology Europe B.V., Frankfurt am Main, Germany

## Data Availability

All data will be shared upon reasonable request.

## Supplementary Materials

The following supporting information can be downloaded at https://www.mdpi.com/article/10.3390/ijms26157033/s1.

## Abbreviations

The following abbreviations are used in this manuscript:

ALDH1	Aldehyde dehydrogenase 1
CSCs	Cancer stem cells
E	Epithelial
EMT	Epithelial-to-mesenchymal transition
GSK-3β	Glycogen synthase kinase 3-β
ILK	Integrin-linked kinase
M	Mesenchymal
MAPK	Mitogen-activated protein kinase
NF-κB	Kappa-light-chain-enhancer of activated B cells
TGF-β	Transforming growth factor-β
