# Supplementary figures and images for: Differential Effects of Snail-KO in Human Breast Epithelial Cells and Human Breast Epithelial × Human Breast Cancer Hybrids
Source: Int J Mol Sci. 2025 Jul 22;26(15):7033. doi: 10.3390/ijms26157033 (PMC12345957; doi:10.3390/ijms26157033)

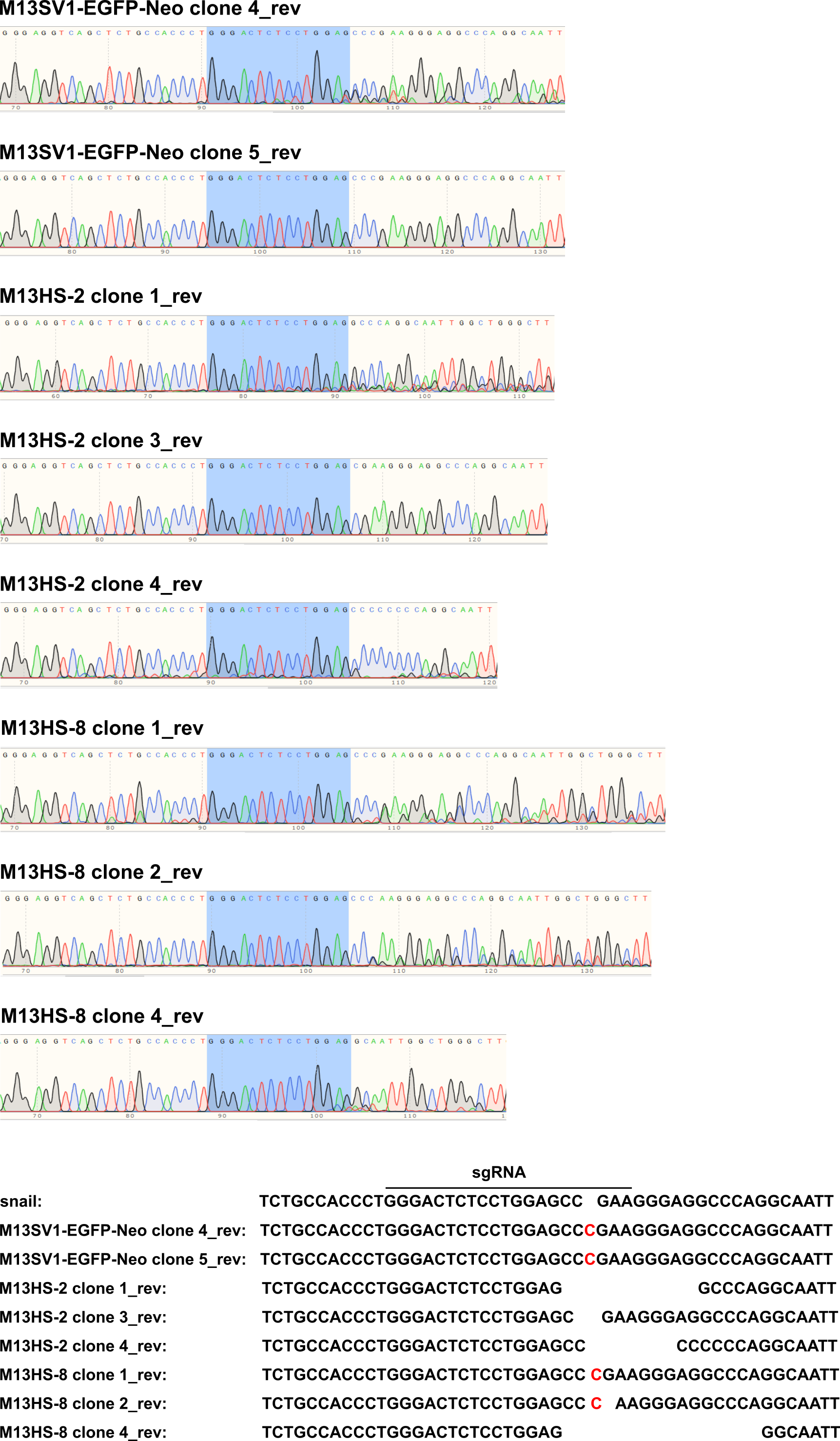

Supplement: Supplementary file 1 [file ijms-26-07033-s001.zip › ijms-3737077-supplementary.png]
